# Union of the American and Southern Dental Associations

**Published:** 1897-04

**Authors:** Thomas Fillebrown

**Affiliations:** Boston, Mass.


### UNION OF THE AMERICAN AND SOUTHERN DENTAL ASSOCIATIONS.

BY THOMAS FILLEBROWN, M.D., D.M.D., BOSTON, MASS.

The American Dental Association was formed as a more perfect organization to succeed the American Dental Convention, which it soon supplanted.

The Southern Dental Association was organized to supply a need which can now be better met by one large truly national body.

Both Associations have done good work, and each has supplied a want existing in the professional life of this nation.

Both have been practically local societies, though ostensibly national. The Southern has been limited by its name and its practice. The American has continued too much an Eastern institution, and has hardly kept pace with the march of progress. Its methods no longer serve its best interests; and there need be no surprise at the loud call that is heard for a reorganization and the adoption of methods that shall infuse new life into its councils.

The vital question to-day is, how to form and organize a society which shall be truly national, and serve the needs of the dental profession of this country.

The most natural course seems to be a union of the American and Southern Dental Associations in one strong, national society, with a constitution which protects all interests, invites the best

---

efforts of all its members, and provides for progress and enlargement in the future.

The profession united in one national association will respect itself more than it can while divided, will be more influential at home, and thus be able to do more to elevate professional character and make the influence of the dental profession in America more potent in all its relations with the world.

The social element is one of the most powerful forces for the progress of humanity, and this will be greatly strengthened by union. Only few men feel able to afford the time, to say nothing of the expense, to attend two series of meetings in one season both serving the same end; hence under the present dual organization many are deprived of the satisfaction and benefit of personal acquaintance. In one association the members from the East, South, and West will come together at regular periods, compare experiences, offer mutual suggestions, and enlarge, deepen, and strengthen their personal relations. All those who were fortunate enough to attend the union meeting of the American and Southern Association in Louisville in 1888 realize how pleasant it is to dwell together in unity.

By union the importance of the State societies will be enhanced; as the meetings of the larger body in each section of the country will be less frequent; the leading men of each State will naturally interest themselves in their home societies and make their proceedings more valuable, and thus be more able auxiliaries to the National Association.

In 1894, at Old Point Comfort, the American Dental Association appointed Thomas Fillebrown, J. Y. Crawford, Louis Jack, B. Holly Smith, and J. N. Crouse a committee to promote the cause of union of the two Associations, and invited the Southern Dental Association to appoint a like committee for the same purpose.

In response to the invitation, the Southern, at its meeting in Atlanta in 1895, chose Drs. L. G. Noel, E. P. Beadles, J. T. Calvert, F. Peabody, and J. R. Knapp a committee to consider the subject with the committee of the American. These committees have sought to obtain a consensus of opinion as to the desirability of union and the essential points of a plan which would prove acceptable to the members of the Associations, which would protect the interests of the minorities, and at the same time provide the best working plan at the present time possible.

At first considerable opposition to the movement was expressed. The opposition seemed based upon the fear that rights would be denied the minority and also upon affection for the old Associations. As the matter has become better understood opinion has been more and more favorable to the movement. The votes of both Associations, although not decisive, have invariably been favorable to a union.

A plan including the following provisions meets with quite general acceptance.

1. It seems desirable to take a new name, one distinctly national; just what it should be has not yet appeared. The committee invites suggestions from any member interested.

2. Divide the country into divisions, South, East, and West, and meet alternately in each section. This will insure a meeting in each portion of the country every third year.

3. Organize the new Association in sections the same as the American is now, but have the president (or executive committee) appoint chairmen known to be competent and interested to do the work. It is believed this would lead to more effective and better work. Often in the past but few would be present at the organization of the sections, and men neither fitted nor interested would be chosen to official positions.

A return to the old committee plan has been sometimes advocated. This plan has been followed in the Southern, and the results of the past few years have not been especially encouraging for its adoption in a new Association.

The plan of sections above proposed contains the essentials of the committee plan, but it makes it obligatory upon every member to join one of them, and insures an efficient head to organize and lead the work.

5. A more intimate relation with the State societies may be promoted by providing that they become practically branches of the National Association, and also by making it the duty of societies sending delegates to make a report of the year's work of the society to the Association.

6. Provide for membership of permanent delegates and honorary members as the American Association does now.

7. Choose a president at large, or from the section in which the last annual meeting was held.

Choose one vice-president from each section, the vice-presidents to be of equal rank, and not first, second, and third, as is now the case in both Associations.

8. To many it seems desirable to change the date of the meeting to some time in September, so that members will come fresh from their vacations ready for work, instead of tired out at the end of an exhausting year. The change would also avoid the excessive heat of August.

While the College Faculties Association and the National Board of Dental Examiners have done a great work for the uplifting of the profession, they have thus far been a direct injury to the interests of the National Associations.

The Southern has suffered because so many of her members have necessarily neglected its meetings to attend the meetings of the Faculties and Board of Examiners which have been held at the same time and place as the meetings of the American. The American has suffered severely by the meetings of the Faculties and Board of Examiners overlapping its meetings and absorbing the attentions of otherwise active members.

This can be remedied in one of two ways, either by the Faculties and Examiners meeting at another time than that of the National Association, or by the meetings being held a week earlier at the same place as the National Association. The latter plan is very likely to be tried the present year, and its effects can be then properly estimated. It is quite reasonable to expect the meeting of the Faculties Association to be called as early as the Friday before the time of the meeting of the American and the Southern Associations at Old Point.

If the Board of Examiners shall also meet early, the work of these bodies will be completed, and the members be left free to engage in the work of the National Association. This is certainly one of the most important considerations for the interests of all concerned.

Dr. W. C. Barrett, at the last meeting of the American Association, and Dr. A. H. Thompson, in an article in a late issue of the *Dental Cosmos*, and several other interested members, have expressed the conviction that division associations should be formed to meet annually as parts of the national society. It is undoubtedly a wise and desirable thing to do, and it seems now quite possible to carry out this idea by providing for it somewhat as follows:

1. The members of each division South, East, and West may form one or more branches to meet annually except the year the National Association meets in the same division.

2. Each branch shall manage its own affairs, subject to the constitution and regulations of the parent society, elect its own officers, and pay its own incidental expenses.

3. Each branch to receive delegates from societies within its limits, and they shall have the same standing in the National Association as those joining direct from local societies.

4. The proceedings of the branches to be sent to the National Association for publication in the transactions of the year.

The details of a plan to accomplish this result can be arranged which will not interfere with the customary working of the National Association. This plan will prevent the destruction of the present societies, and thus remove the principal objection that has been raised against union. One branch would be practically the Southern Association, and the Eastern would include a large proportion of the American membership.

The West might reasonably be able to form two branches. The Western branch and the Pacific branch. Eventually the best interests of the profession may be served by making four divisions of the country. At present we shall probably better succeed with three.

This article does not presume to be exhaustive, but is only suggestive.

The discussion of the subject will show us the best way.

The committee has no plan nor desire save to formulate the wishes of the members of the two associations and invite suggestions as to the points mentioned, or any others which it seems desirable to have considered.

